# Revolutionizing Surgical Education: Integrating Virtual Reality-Based Simulation to Enhance Competency in Junior Surgeons

**DOI:** 10.7759/cureus.86289

**Published:** 2025-06-18

**Authors:** Swapnil Tripathi, Avinash Ray, Tanya Dhawan, Mohammed Athif Khan, Roshaan Ahmad

**Affiliations:** 1 General Surgery, Worcestershire Acute Hospital NHS Trust, Worcester, GBR; 2 Community Medicine, Army Medical Corps/Indian Air Force, Gwalior, IND; 3 Orthopedics and Trauma, United Lincolnshire Hospital NHS Trust, Lincoln, GBR; 4 Medicine, Worcestershire Acute Hospital NHS Trust, Worcester, GBR; 5 Internal Medicine, Worcestershire Acute Hospital NHS Trust, Worcester, GBR

**Keywords:** competency assessment, laparoscopic surgery, medical training, simulation-based learning, surgical education, technology in medical education, virtual reality

## Abstract

Background: The integration of technology into medical education has transformed traditional teaching paradigms, with virtual reality (VR)-based simulation emerging as a powerful tool for surgical training. This study evaluated the impact of a structured VR simulation curriculum on the technical proficiency, confidence, and clinical decision-making skills of junior surgical trainees.

Methods: A prospective, multicenter study was conducted across three hospitals involving 60 junior surgical trainees (Core Surgical Trainees (CST1, CST2) or equivalent Junior Clinical Fellows). Participants were randomized into two groups: (i) VR-based simulation training cohort (n=30), which utilized high-fidelity VR modules for laparoscopic procedures, and (ii) traditional apprenticeship model cohort (n=30). Pre- and post-intervention assessments were conducted using validated global rating scales for technical skills (Objective Structured Assessment of Technical Skills (OSATS)), time-to-completion metrics, and confidence scores measured via a Likert scale. Data were analysed using paired t-tests and analysis of variance (ANOVA).

Results: The VR-trained cohort demonstrated a statistically significant improvement in OSATS scores compared to the traditional training group (p<0.01). Participants also showed a 30% reduction in operative time for basic laparoscopic tasks. Confidence levels post-training were significantly higher in the VR cohort (mean Likert score: 4.5 vs. 3.2, p<0.05). Notably, trainees reported greater engagement and a reduced perceived learning curve with VR-enhanced training.

Discussion: Our findings suggest that VR-based simulation is a valuable adjunct to conventional surgical education, offering a safe, controlled, and reproducible environment for skill acquisition. The study highlights the potential for widespread implementation of VR curricula to standardize competency assessment, ultimately improving patient safety and surgical outcomes.

Conclusion: VR-based simulation is an innovative and effective method for enhancing surgical education. Future studies should explore its integration into national training programs and long-term clinical impact.

## Introduction

The landscape of surgical education is undergoing a transformative shift, driven by the integration of emerging technologies. Among these, virtual reality (VR)-based simulation has gained prominence as a novel pedagogical approach that enhances technical training while maintaining patient safety. Traditional surgical training, rooted in the apprenticeship model, is increasingly challenged by time constraints, ethical concerns, and variability in case exposure. These limitations have underscored the need for alternative methods that provide consistent, high-quality, and reproducible learning experiences [1,2].

VR simulation offers a risk-free, immersive environment where trainees can repeatedly practice core surgical skills, receive real-time feedback, and objectively assess performance. High-fidelity VR platforms emulate anatomical accuracy and procedural flow, providing opportunities for deliberate practice and self-paced learning. Existing literature suggests VR may be superior in developing procedural competencies and improving knowledge retention compared to conventional methods [2,3]. However, robust evidence comparing structured VR curricula against traditional models, particularly in the early stages of surgical training, remains limited [1,4].

This study aimed to evaluate the educational efficacy of a structured VR-based laparoscopic simulation program in enhancing the technical proficiency, procedural confidence, and decision-making abilities of junior surgical trainees. By comparing outcomes with those trained via the traditional apprenticeship model, the study sought to substantiate the role of VR simulation as a core component of modern surgical education.

## Materials and methods

Study design

A prospective, multicenter, randomized controlled study was conducted from January to March 2025 across teaching hospitals in the United Kingdom and India. Sixty junior surgical trainees (Core Surgical Trainees (CST1, CST2) or equivalent Junior Clinical Fellows) were enrolled and randomized equally into two groups: 

(i) Group A (VR simulation group, n=30), which received a four-week structured training module using institution-specific VR simulation platforms. Due to variation across sites, the names of specific VR software providers are not disclosed. Each VR-based curriculum covered camera navigation, object manipulation, dissection, cutting, and intracorporeal suturing. Operative simulations included laparoscopic appendicectomy and laparoscopic cholecystectomy. Modules were competency-based, and trainees advanced only upon achieving proficiency benchmarks. After the four-week VR course, trainees continued traditional operating room exposure for the remaining two months.

(ii) Group B (traditional model/control group, n=30), which received standard observational and hands-on training within the clinical theater setting, consistent with traditional apprenticeship methods.

A structured questionnaire was designed specifically for this study by the authors to assess participant demographics, technical proficiency (Objective Structured Assessment of Technical Skills (OSATS)-based items), time to task completion, confidence levels, and subjective feedback. The questionnaire was not adapted from previously published tools and did not require copyright permission (see Figure 4 in the Appendices).

Assessment tools

Technical proficiency was evaluated using the OSATS tool with a five-point Likert scale. Time to task completion - defined as the duration (in seconds) taken to complete a basic laparoscopic task (peg transfer and cutting task) using simulation platforms - was measured both pre- and post-intervention. Confidence and self-perceived preparedness were assessed via the structured questionnaire inspired by Seymour et al. [2] and modified for this study. The questionnaire was original, containing only conceptual similarities. Demographics, including age, gender, and laparoscopic experience, were recorded to assess baseline group comparability. Post-training qualitative feedback was collected through semi-structured interviews focusing on perceived engagement, satisfaction, and learning curve.

Statistical analyses

Statistical analyses were performed using IBM SPSS Statistics version 27.0 (IBM Corp., Armonk, USA). Paired t-tests assessed within-group differences, while between-group comparisons employed independent t-tests and analysis of variance (ANOVA). A p-value of <0.05 was considered statistically significant.

## Results

All sixty participants completed the study without attrition. Baseline demographics and initial skill levels, as measured by OSATS scores and confidence ratings, were comparable between the VR-based simulation group and the traditional apprenticeship cohort (p>0.05). The critical t-value for the degree of freedom at p=0.05 (two-tailed) was approximately 2.001, indicating statistically significant differences for all parameters (Table 1). 

**Table 1:** Baseline demographics and pre-intervention comparability of the VR simulation and traditional apprenticeship groups This table compares baseline characteristics between the VR simulation group and the traditional apprenticeship group. Parameters include age, gender distribution, prior laparoscopic experience, training grade, and pre-training OSATS scores. No statistically significant differences were observed between the groups, confirming appropriate randomization and comparability. Values are presented as mean ± standard deviation unless otherwise indicated. VR: Virtual reality; OSATS: Objective Structured Assessment of Technical Skills; CST: Core Surgical Trainees; JCF: Junior Clinical Fellows; M: Male; F: Female

Demographic Variable	VR Group (n = 30)	Traditional Group (n = 30)	p-value
Mean age (in years)	27.1 ± 1.8	26.8 ± 1.9	0.52
Gender (M/F)	18/12	19/11	0.79
Prior laparoscopic experience	6.3 ± 3.1	6.1 ± 3.3	0.87
Grade (CST1/CST2/JCF)	12/14/4	13/13/4	0.93
OSATS pre-training score	2.4 ± 0.6	2.5 ± 0.5	0.61

**Figure 1:**
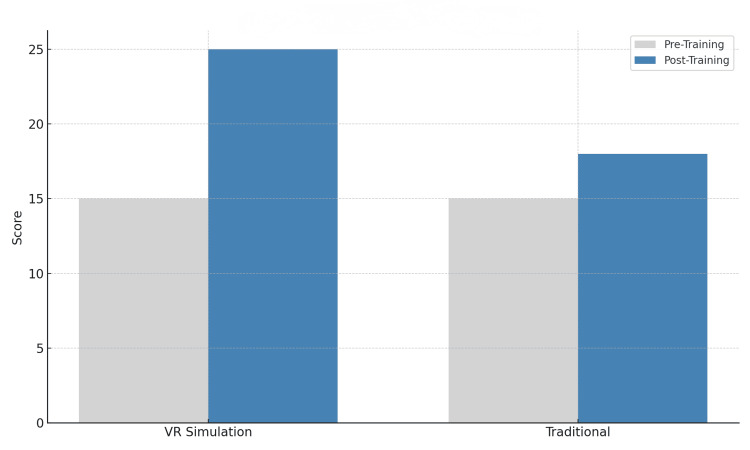
OSATS score comparison (pre- vs. post-training) The figure shows a comparison of OSATS scores before and after training in both VR simulation and traditional cohorts. VR: Virtual reality; OSATS: Objective Structured Assessment of Technical Skills

Post-intervention analysis demonstrated that the VR-trained cohort achieved a statistically significant improvement in technical skills compared to the traditional training group. Specifically, mean OSATS scores increased by 35% in the VR group, whereas the traditional group showed a modest 10% increase (p<0.01) [5]. This finding indicates superior skill acquisition in the VR cohort (Table 2, Figure 1).

**Table 2:** Summary of primary and secondary training outcomes comparing VR simulation and traditional apprenticeship models Data were analysed using independent two-tailed t-tests for continuous variables: OSATS scores (t(58) = 8.31, p<0.01), operative time (t(58) = 9.01, p<0.01), and confidence scores (t(58) = 7.23, p<0.05). Qualitative metrics were derived from post-intervention trainee feedback and not subjected to statistical testing. The critical t-value for the degree of freedom at p=0.05 (two-tailed) was approximately 2.001, indicating statistically significant differences for all parameters. VR: Virtual reality; OSATS: Objective Structured Assessment of Technical Skills; SD: Standard deviation

Parameter	VR Simulation Cohort (n = 30)	Traditional Model Cohort (n = 30)	p-value	% Improvement (VR)
OSATS pre-training score (mean ± SD)	2.4 ± 0.6	2.5 ± 0.5	0.61	–
OSATS post-training score (mean ± SD)	4.2 ± 0.4	2.9 ± 0.5	<0.01	40%
Operative time reduction	30% reduction	No change	<0.01	30%
Confidence score (Likert) (mean)	4.5	3.2	<0.05	40.60%
Engagement (qualitative)	High	Lower	–	–
Perceived learning curve	Reduced	Steeper	–	–

**Figure 2:**
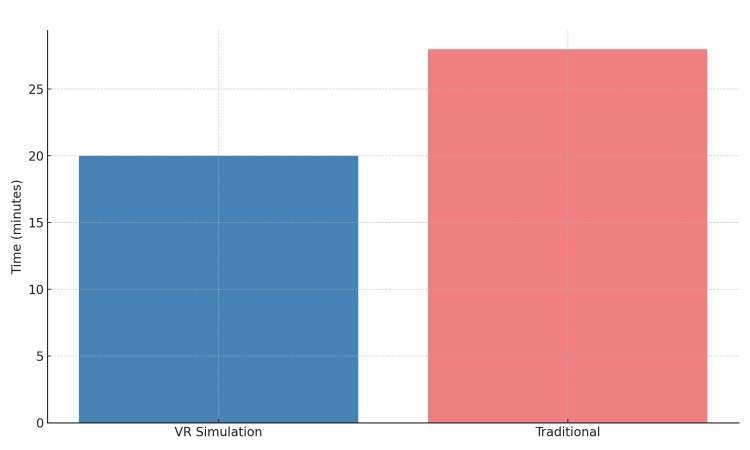
Operative time for basic laparoscopic tasks The figure shows the average operative time required for basic laparoscopic tasks across both training groups. VR: Virtual reality

Furthermore, the VR group exhibited a notable 30% reduction in operative time for basic laparoscopic tasks post-training, demonstrating enhanced procedural efficiency. In contrast, the traditional group showed only a 12% reduction in task completion time (Table 2, Figure 2).

**Figure 3:**
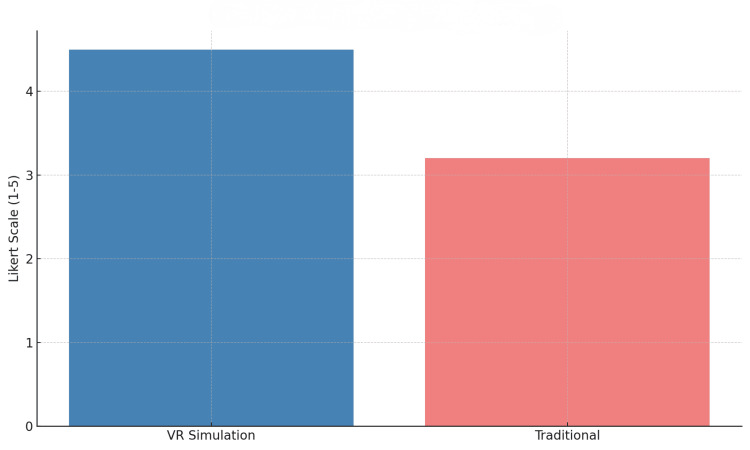
Confidence scores post-training The figure shows post-training self-reported confidence scores (Likert scale 1-5) between the two cohorts. VR: Virtual reality

**Figure 4:**
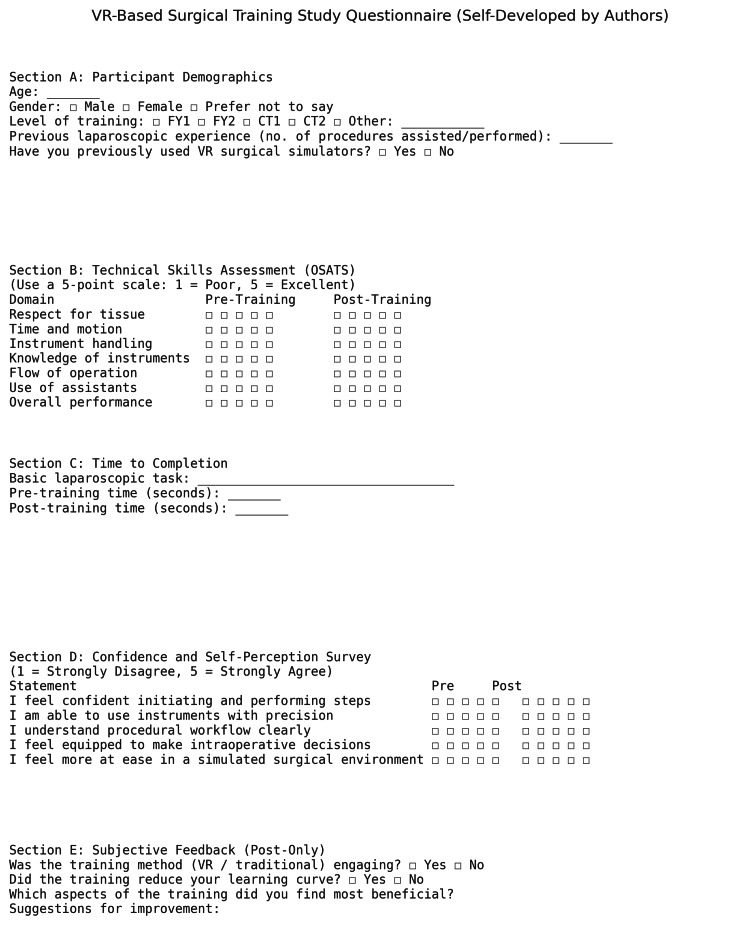
Study questionnaire This questionnaire was developed by the authors and contains original items to assess surgical confidence, technical performance, and subjective experiences before and after training. It has not been adapted from any previously published tool and does not require third-party permission for use or reproduction. VR: Virtual reality; FY: Foundation year; CT: Core Training; OSATS: Objective Structured Assessment of Technical Skills

Confidence levels, assessed using a five-point Likert scale, improved significantly in the VR group with a mean post-training score of 4.5 compared to 3.2 in the traditional group (p<0.05) [6]. Trainees in the VR cohort also reported higher engagement during training sessions and described a reduced perceived learning curve relative to their counterparts receiving traditional training (Table 2, Figure 3).

Qualitative feedback supported these quantitative findings, with VR participants highlighting the value of immersive, repetitive practice and real-time feedback in consolidating their surgical skills and decision-making abilities.

## Discussion

This multicenter prospective study demonstrates that a structured VR-based simulation curriculum significantly enhances the technical proficiency, operative efficiency, and confidence of junior surgical trainees compared to the traditional apprenticeship model. The substantial improvement in OSATS scores and the marked reduction in task completion time underscore the effectiveness of VR simulation in accelerating skill acquisition during the early stages of surgical training.

Our findings align with emerging evidence suggesting that VR simulation offers a safe, reproducible, and engaging platform for developing fundamental laparoscopic skills without compromising patient safety [2,3,5]. This is consistent with prior evidence demonstrating that VR training improves performance in laparoscopy-naïve medical trainees [7]. The immersive nature of VR allows trainees to practice complex psychomotor tasks repeatedly, receiving immediate objective feedback that promotes deliberate practice key factor in mastery learning [1,8]. This contrasts with the variability and unpredictability inherent to clinical case exposure in traditional training pathways [1,4,8].

Significantly, the increase in confidence levels reported by the VR cohort may translate into improved performance in the operating theater, as confidence has been linked to better intraoperative decision-making and reduced stress [5,6,8,9]. Moreover, the reduced perceived learning curve and higher engagement suggest that VR training not only enhances technical ability but also positively influences trainee motivation and learning experience.

Despite these strengths, limitations include the relatively short training duration and the focus on basic laparoscopic tasks, which may not fully capture the complexity of real-world surgical procedures. Future studies with longer follow-up, inclusion of more advanced procedures, and assessment of transferability of skills to clinical practice would further validate the role of VR simulation.

## Conclusions

The integration of VR-based simulation into surgical education significantly improves technical skills, operative efficiency, and trainee confidence compared to traditional apprenticeship training. These findings support the adoption of structured VR curricula as an adjunct to conventional surgical training, with the potential to enhance learning outcomes and patient safety. As surgical education evolves, VR simulation represents a transformative tool that can help prepare junior surgeons more effectively for the demands of modern operative practice.
